# Design of freeform mirrors using the concentric rings method

**DOI:** 10.1016/j.heliyon.2023.e14229

**Published:** 2023-03-03

**Authors:** Jorge González-García, Agustin Santiago-Alvarado, Angel S. Cruz-Félix

**Affiliations:** a Physics and Mathematics Institute, Technological University of the Mixteca, Carretera a Acatlima km 2.5, Huajuapan de León, Oaxaca C. P. 69000, Mexico; b Postgraduate Studies Division, Technological University of the Mixteca, Carretera a Acatlima km 2.5, Huajuapan de León, Oaxaca C. P. 69000, Mexico; c Optics Coordination, National Institute of Astrophysics, Optics and Electronics, Luis Enrique Erro No.1, Sta. Ma. Tonantzintla, Puebla C. P. 72840, Mexico

**Keywords:** Freeform surface, Segmented mirror, Concentric rings method

## Abstract

Through the methodology of optical surface design based on concentric rings, this paper proposes the design of freeform mirrors, initially by employing segmented rings, each of them with different spherical radii of curvature, and then by employing segmented conic rings with different conic constants in each of the segments. These surfaces will then produce the desired images. For the case of segmented spherical rings, mathematical expressions were deduced to obtain the image points as a function of the radii of curvature. Furthermore, it is shown that in the case where conic rings were used, there is a decrease in spherical aberration, which allows the manipulation of the generated image. Finally, several proposals are presented for the design of mirrors to generate both the desired size of the image and the desired distribution of energy, together with their analyses.

## 1 Introduction

Developing optical devices has always represented a significant challenge since fulfilling the objectives with the desired requirements and performance at a low cost is not always possible. The design and production of optical systems has changed over time, mainly due to advancements in innovation and technology, from the use of optical components with spherical and plane profiles to more complex surfaces such as conics, Fresnel, toroidal, and most recently, freeform surfaces, such as the ones used in telescopes, illumination and concentration systems [1,2]. Furthermore, the materials employed in the manufacture of these components have included metal, glass, carbon fiber, and polymers with reflecting film, etc. [3].

A common approach to the design of a component starts with an initial design being able to reduce a certain amount of optical aberrations. From this, convergence must be achieved to a well-corrected minimum in the optimization process [4]. It is well known that freeform surfaces make use of many variables in the optimization process, which is one of the reasons they are considered.

Some of the current optical design software programs have incorporated aspheric surfaces, tolerance measuring tools, and even the use of freeform surfaces in their libraries. However, there should be some degree of caution when using this software due to the various engineering challenges in their development and understanding of appropriate theories, optimization techniques, mathematical descriptions, and their application in manufacturing and testing methods [5].

There is no unique methodology for designing all freeform surfaces, as this depends on the specific application [6]. Various methods for the design of freeform surfaces are found in the literature, such as the direct method [[7], [8], [9]], simultaneous multiple surfaces (SMS) [6,7,[10], [11], [12]], point-to-point mapping, through the Monge-Ampère differential equation [13,14], the construction-iteration method [15], quadric [12,16], feedback [12], NURBS [6,17], through the Wassermann-Wolf differential equation, geometric and variational adaptation [6,[18], [19], [20], [21]], Fermat's [7,18,19], two grids of equi-spatial equi-flow [14], automated from an initial design [7,22], description and reimplementation of real freeform surfaces, vectorial aberration [4], field extension construction [8], through the perturbing coaxial system, field or aperture, and, union and fusion [9], among other techniques.

Each method adopts a particular mathematical description of the freeform surface, which may be local or global, and they implement either radial basis, splines, wavelets, or hybrid functions. With this approach, common choices for the global description are the set of Zernike and 2D-Q polynomials, and non-orthogonal bases such as X–Y polynomials may still be used. Furthermore, the set of Chebyshev polynomials and the nodal aberration theory have recently been employed to analyze off-axis and misaligned systems [5,7,10,17].

Regarding the mathematical description, the following models are found for the design of freeform surfaces: (i) X–Y polynomials: non-orthogonal basis and they cannot be well-adapted to circular shapes which results in the inclusion of an unnecessary compensatory term [6,13,17,23]; (ii) Zernike polynomials: not ideal for optimization if the freeform surface is located at a long distance from the system pupil or if the pupil does not have a circular shape [7,13,17,23]; (iii) φ-polynomials: they differ from the original set of Zernike polynomials in their scheme configuration and they are helpful for applying the design in manufacturing [13]; (iv) Q-polynomials: the description of the freeform surface is related to the surface sag through the polynomial coefficients [13,17,24]; (v) anisotropic radial basis functions: the main benefit of implementing these surfaces is that they compare well with other local and selected global representations and are not restricted by a domain shape [25].

On the other hand, optical systems are classified regarding their applications as imaging systems, illumination systems, and light concentration systems, or regarding their symmetry as systems with two orthogonal symmetry planes, systems with a symmetry plane, non-rotational symmetric systems, and asymmetric systems [7].

Optical designers face several challenges when designing an optical system. In the case of imaging systems designed to reduce optical aberrations [13,26], they begin with a start design or initial plane to optimize with freeform surfaces [13,15,27], or they make use of the nodal aberration theory for tilted, decentered and freeform systems [13]. The obstacles that need to be overcome include the high degrees of freedom and the interpretation and control of aberrations since the complexity of the merit function is increased, meaning that new design methods for optimizations must be developed.

In the case of illumination systems, radiation transfer needs to be optimized, and an integral methodology is often required [13,19,24]. However, other methods are implemented in specific cases, such as the brute force method, the Monge-Ampère differential equation, the Mapping method [13,14,16,28], and the inverse problem or tailoring [16,28].

Moreover, the development of special algorithms for freeform illumination systems allows the design of one or several freeform surfaces so that the light emitted from a source is redirected to produce the desired illumination [12].

Finally, regarding the design of light concentration systems, the designer must contemplate several variables, such as the desired shape of energy concentration, the concentration ratio, the power of concentration, the radiation flux uniformity, the optical performance, the amount of aberrations, the material and shape of the reflector, the intensity of solar radiation, the dish diameter, the aperture area, and the focal length [[1], [2], [3]], among others. Other factors to consider include the ensemble components used for construction and the type of the collector, such as parabolic trough, dish, tower, Fresnel, as well as facets, either in one piece or segmented [2,3,29]. The design methods used for these systems are the same as those used in illumination systems. In addition, a method is found that uses a dish-type comprised of concentric rings to generate the desired spot size [30,31].

This work presents a design methodology for the development of freeform or aspheric optical systems based on a dish-shaped mirror formed by segmented concentric rings, which will allow the formation of a specific and desired image as well as the desired areas of radiation concentration. Such a method can be used to design several optical instruments by the adequation of an optimization function as a sole requirement. In the following sections, the problem to solve and the design of symmetric and asymmetric mirrors are described in order to generate the desired spot size and specific shapes. Finally, a discussion and conclusions are presented.

## 2 Design methodology

Several proposals for lens designs can be found in the literature where the concept of surfaces comprised of spherical concentric rings has been employed, i.e., each ring has a different radius of curvature, and they can generate the desired image at a specific location [30]. Furthermore, this approach is also used in the design of mirrors to generate the desired spot size for solar concentration applications [30,31]. In this direction, and with this concept in mind, a design method for aspheric or freeform concave mirrors is proposed, where its surface is comprised of a set of segmented spherical concentric rings, with each segment having a different radius of curvature.

**Fig. 1:**
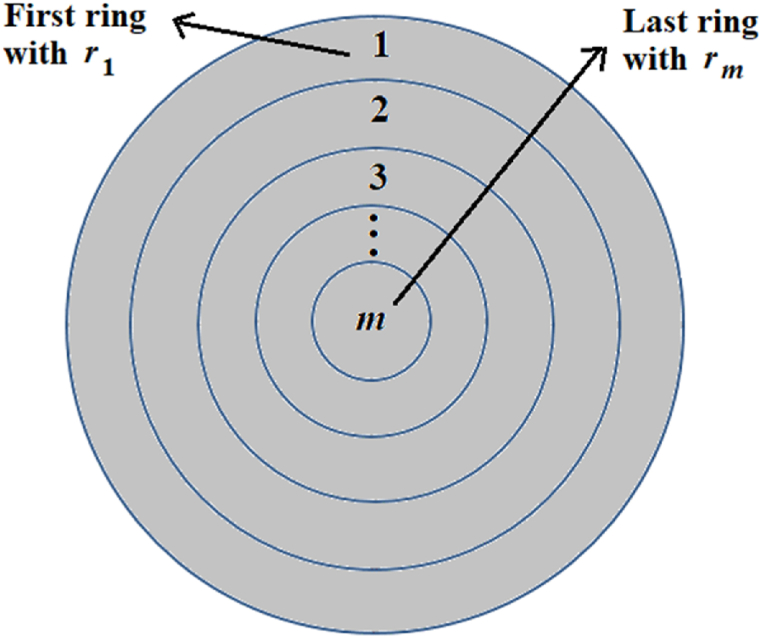
Diagram of a mirror design with a surface comprised of a set of *m* spherical rings.

The proposed methodology will be applied to the design of a dish-type mirror, considering that its surface consists of a set of *m* spherical rings with different radii of curvature *r*_*m*_, as shown in Fig. 1.

**Fig. 2:**
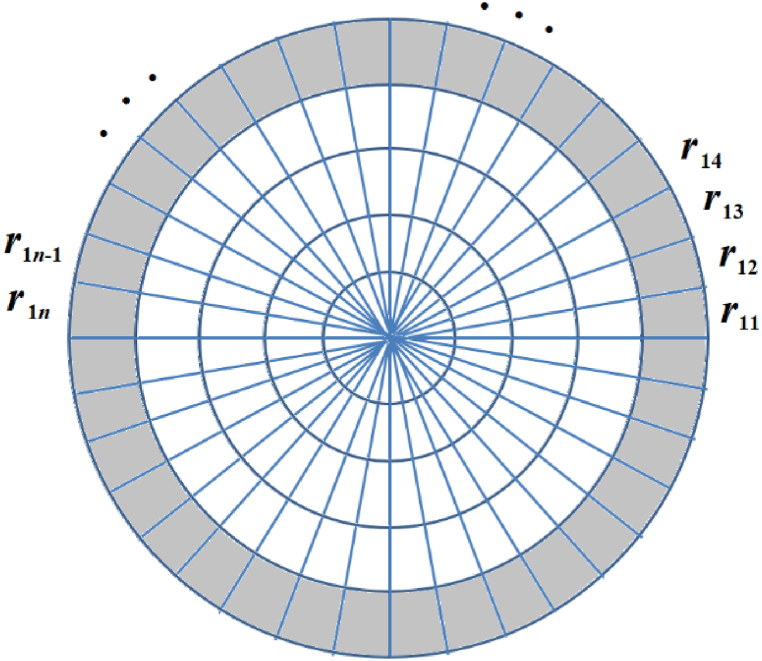
The mirror surface design comprises *m* spherical rings, and each ring is formed by *n* segments, with each segment having a different radius of curvature.

A stage of optimization is applied to the start design considering two specific cases. The first case considers that the radii of curvature of the rings is variable to obtain the design of aspheric surfaces which will result in a mirror that generates a desired size of an image. The second case contemplates a stage of optimization where each ring is comprised of a set of segments (see Fig. 2), and each segment has a different radius of curvature in order to obtain the design of freeform surfaces. That is to say, a mirror design with non-rotational symmetry that generates images with a desired non-rotational shape. An exact ray trace was performed in order to achieve this [32]. Fig. 2 shows the terminology that was followed for the description of the segments, with each ring comprised of *n* segments.

General ray-tracing equations for spherical surfaces were implemented to the start design for both non-segmented rings and segmented rings [32]. In the case of a mirror ray tracing, after algebraic manipulation of the equations, a set of equations was derived that provided the coordinates of the image points (defined as the $\left({zs}_{i},{yz}_{i}\right)$ coordinates in the image plane of the simulated image or generated image, whose coordinates system was defined in each plane according to the exact ray tracing method [32]) through the initial parameters of the optical system, given by:

(1) ysi=yi+(1−c12ρ2)12(−2c1yit1+2c12ρ2yi)+2c13ρ2yit1−2c1yit1+4c14ρ4yi+6c15ρ4yit1(1−c12ρ2)12(1−2c12ρ2)+1−2c12ρ2,

(2) zsi=zi+(1−c12ρ2)12(−2c1zit1+2c12ρ2zi)+2c13ρ2zit1−2c1zit1+4c14ρ4zi+6c15ρ4zit1(1−c12ρ2)12(1−2c12ρ2)+1−2c12ρ2,

where, $\left(z_{i},y_{i}\right)$ are the coordinates in the reference plane which are known parameters; $\rho =\sqrt{z_{i}^{2}+y_{i}^{2}}\text{,}$ is the radial parameter; *c*_*1*_ is the center of curvature of the mirror and is related to the radius of curvature by $c_{1}=1/r\text{;}$ $t_{1}$ is the mirror vertex distance to the simulated image and is also a known parameter. When the mirror is comprised by a set of *m* rings, equations (1), (2) must consider the definition: $c_{1j}=1/r_{j}\text{,}$ with j=1,2,...,m.

**Fig. 3:**
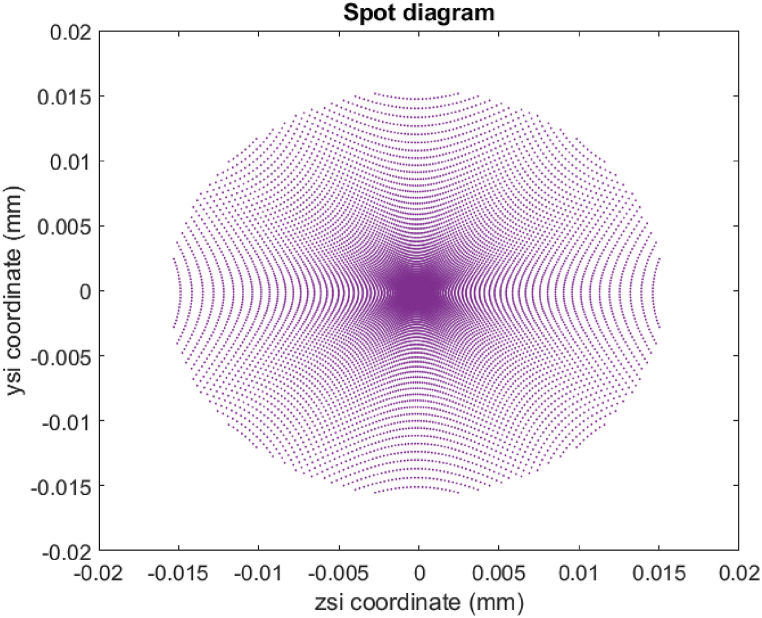
Image from a point source generated by a spherical mirror at a distance $t_{1}$ from the vertex.

Fig. 3 shows the spot diagram generated through equations (1), (2) considering a conventional spherical mirror with a diameter of 100 mm and a 1040 mm radius of curvature. The generated image corresponds to a point source located at infinity. The image generated by this mirror is set up as the desired image to be reproduced by the designed mirrors comprised of rings (non-segmented and segmented). The variation in the diameter of the designed mirror is useful for generating the desired image in its original and smaller size. In the following section, several examples that illustrate the potential of this methodology are presented.

## 3 Design of mirrors

### 3.1 Rotational-symmetric mirror

**Fig. 4:**
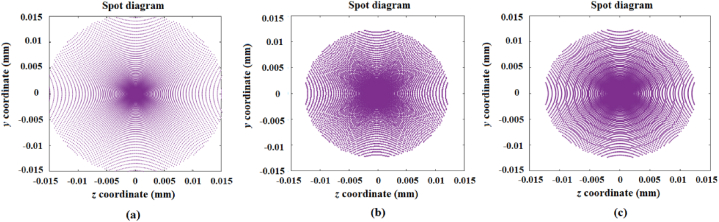
a) Image generated by a reference mirror with a 100 mm diameter and a radius of curvature of 1040 mm; b) image from the reference mirror on a 1 to 0.8 scale; c) image generated by the designed mirror with a surface comprised of a set of 20 rings.

For the sake of comparison, Fig. 4a shows the image produced by a spherical mirror with a 100 mm diameter and a 1040 mm radius of curvature, generated at 1020 mm from the mirror vertex. Fig. 4b shows the image from the reference mirror on a 1 to 0.8 scale, and this image is considered to be the desired image. Fig. 4c shows the image generated by the designed mirror with a surface comprised of a set of 20 spherical concentric rings where the radii of curvature of the rings were optimized with the aid of the Genetic Algorithms [30,31].

### 3.2 Non-rotational symmetric mirror

As previously mentioned, a mirror with segmented rings becomes a freeform mirror, and it will produce non-rotational symmetric images. In these types of designs, the number of variables to be optimized by the Genetic Algorithms will be increased by a factor equal to the number of segments in each ring- This means that the number of variables to be optimized is obtained as the product of the number of rings by the number of segments.

Consequently, for the first design, a mirror with a surface comprised of a set of 10 rings with each ring divided into 36 spherical segments was considered. The first two outer rings were optimized to obtain a non-rotational symmetric pattern on the periphery of the generated image. With this, the optimized number of variables is decreased, meaning that 72 spherical radii of curvature were optimized. This is considered a practical design, given that the external energy is concentrated in a smaller area.

As a part of the optimization stage, a recurrence relation is implemented as it optimizes all of the 72 variables. However, only ten variables were directly optimized with the aid of the genetic algorithms [30,31]. That is to say, in order to reduce the number of variables to be optimized from 72 to 10, a recurrence formula was used to calculate the 72 curvature radius values. The recurrence equation for the 72 curvature radii is set as

(3) $$r_{k+1}=r_{k}+\Delta r_{j}\text{,}$$

where $r_{k}\text{,}$ with *k* = 1, …, 72, are the curvature radii and $\Delta r_{j}\text{,}$ with *j* = 1, …, 10, are the curvature radius increments. These increments are the variables to be optimized by the Genetic Algorithm and are defined by the following equation

(4) $$\Delta r_{j}=\left(\begin{array}{l} m_{1}\;\text{if}\;1\leq k\leq 7\\ m_{2}\;\text{if}\;8\leq k\leq 14\\ \vdots \\ m_{10}\;\text{if}\;64\leq k\leq 72 \end{array}\right)\text{.}$$

Therefore, $\left({zs}_{i},{ys}_{i}\right)$ are defined as the coordinates of the Simulated Image (*SI*) on the image plane, and are a function of the increments defined by Eq. (4), so that

(5) $$SI\left(z_{i},y_{i},m_{1},m_{2},...,m_{10}\text{,}\right)=\left(zs_{i},ys_{i}\right)\text{,}$$

where, as mentioned above, $\left(z_{i},y_{i}\right)$ are the coordinates of the reference plane, as stated in the exact ray tracing method [32].

Once the coordinates of the desired image $\left(zd_{i},yd_{i}\right)$ are determined, then the fitness function $S^{2}$ can be defined as

(6) $$S^{2}=\sum_{i=1}^{N}{\left(zs_{i}-zd_{i}\right)}^{2}+{\left(ys_{i}-yd_{i}\right)}^{2}\text{,}$$

where *N* is the number of points considered in the simulation. Then, with the description of the coordinates of the simulated image by means of Eq. (5), and the fitness function by means of Eq. (6), the process of optimization can be initiated.

**Fig. 5:**
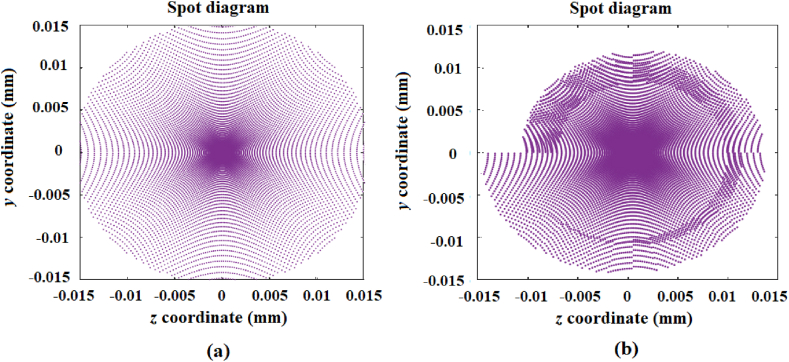
a) Image generated by a reference mirror with a diameter of 100 mm and a 2040 mm radius of curvature; b) image generated from the designed mirror comprised of 10 rings, with each ring divided into 36 spherical segments and the two outer rings optimized.

Again, Fig. 5a shows the image generated by a spherical reference mirror with a 100 mm diameter and a curvature radius of 2040 mm. Fig. 5b shows the generated image with non-rotational symmetry from the designed mirror with a surface comprised of a set of 10 rings, with each ring divided into 36 spherical segments and the two outer rings optimized. All the radii of curvature of the rest of the rings 3 to 10 were considered to be from a spherical mirror with a 100 mm diameter and a radius of curvature of 2040 mm. It can also be seen from Fig. 5b that a spiral pattern is formed due to the decrease in the radii of curvature among the adjacent segments.

### 3.3 Design of circular mirrors for generating images similar to those from a trough-type mirror

In this case, the design of a mirror with a surface comprised of a set of 10 rings was considered, from which the first five were divided into 36 segments, while the other five were left unsegmented with a radius of curvature of 2040 mm. The main purpose of this design was to show that a dish-type or circular mirror is able to generate a similar image as those generated by a through-type mirror. The radii of curvature of the segments have a variation in such a way that the size of the generated image in the vertical direction is, in size, close to one-tenth of the image generated by a reference mirror.

**Fig. 6:**
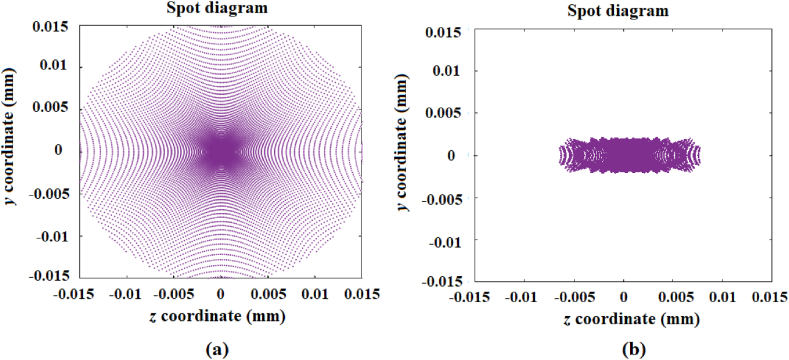
a) Image generated by a reference mirror with a diameter of 100 mm and a 2040 mm radius of curvature; b) image generated from the designed mirror comprised of a set of 10 rings, from which the first outer five are divided into 36 segments.

For the sake of comparison, Fig. 6a shows the generated image from a spherical reference mirror with a 100 mm diameter and a 2040 mm radius of curvature. Fig. 6b shows the image generated by the designed freeform mirror that is comprised of a set of segmented concentric rings, and it can be observed that they tend to be similar to the images produced by a through-type mirror that are employed for the concentration of solar energy.

**Fig. 7:**
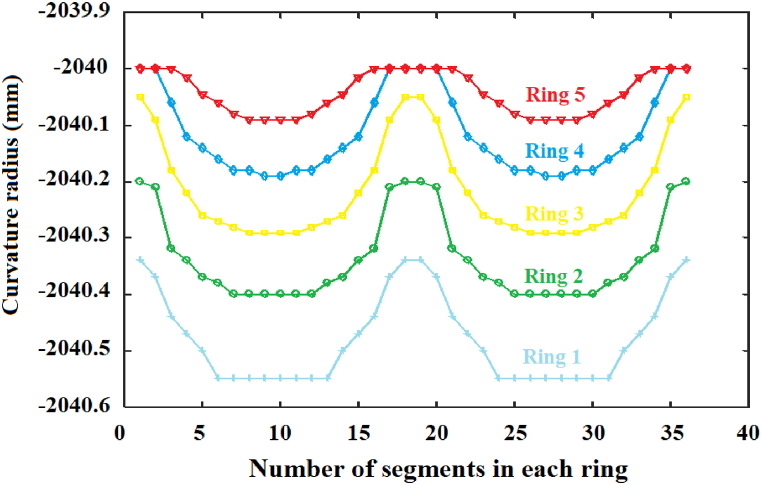
Curves relating to the radii values of curvature from the first five segmented rings in function of the number of segments.

Fig. 7 shows the curvature radii values in function of the number of segments of each ring. Each curve shows the behavior relating to the radii values of curvature from the first five segmented rings that were optimized in order to generate the image shown in Fig. 6b. These curves show that the tendency of the behavior of the segments from each ring is related to the non-symmetric generated image, i.e., tendency curves may be proposed to generate different forms of light concentration.

### 3.4 Designs of mirrors with fixed diameter to generate the desired image sizes as those from mirrors with smaller diameters – segments with a spherical profile

This section presents mirrors designed with a fixed diameter to generate the desired image sizes similar to those produced by spherical mirrors with smaller diameters. It is important to mention that the abovementioned design was generated by selecting the radii of curvature that conveniently generated the corresponding images. That is to say, the optimization method was not applied. However, the implementation of the process for the proposed method was shown, and the generated images are now to be considered as desired images to be generated by mirrors with larger diameters located at different positions or to generate the scaled images. Consequently, the optimization method based on genetic algorithms has been applied to the following proposed designs.

**Fig. 8:**
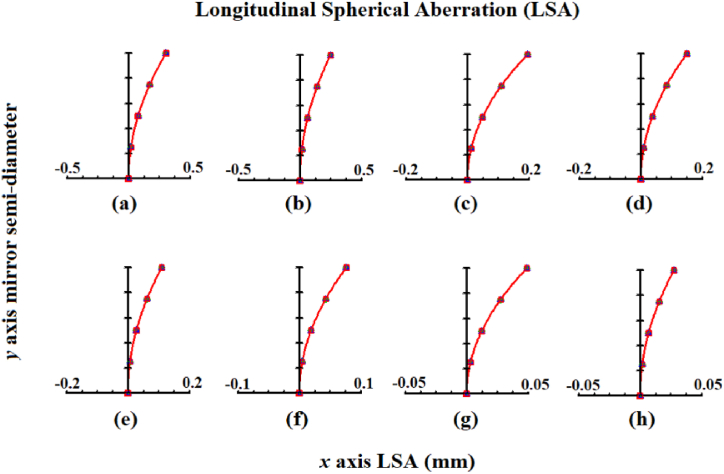
Longitudinal spherical aberration curves for mirrors with diameter: a) 100; b) 90; c) 80; d) 70; e) 60; f) 50; g) 40 and h) 30 mm.

In this following design, the diameter of a spherical mirror is reduced, meaning that its curvature radius is constant (2040 mm), and therefore, a decrease in longitudinal spherical aberration (LSA) is apparent. In Fig. 8, the LSA curves for mirrors with different diameters ranging from 100 mm (Fig. 8a), 90 mm (Fig. 8b), 80 mm (Fig. 8c), 70 mm (Fig. 8d), 60 mm (Fig. 8e), 50 mm (Fig. 8f), 40 mm (Fig. 8g) to 30 mm (Fig. 8h) are shown, and from which a reduction in LSA when the mirror's diameter decreases can be observed.

In the case of the irradiance behavior, as the mirror's diameter is reduced, the irradiance increases considering that the average power remains constant, e.g., when the mirror's diameter is reduced by half, the generated image's irradiance increases by a factor of four. This means that the proposed designed mirrors from this section tend to concentrate more energy in a smaller area.

**Fig. 9:**
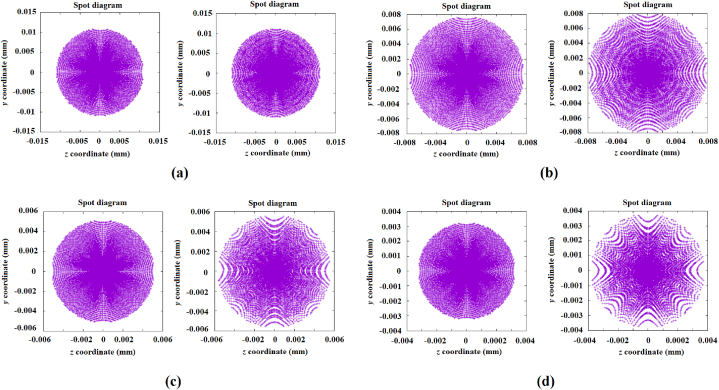
Comparison of the desired images (left column) with the generated images reproduced by the designed mirrors (right column) with segmented rings with spherical profiles. The desired images correspond to the mirrors with diameters: a) 90, b) 80, c) 70, and d) 60 mm.

**Fig. 10:**
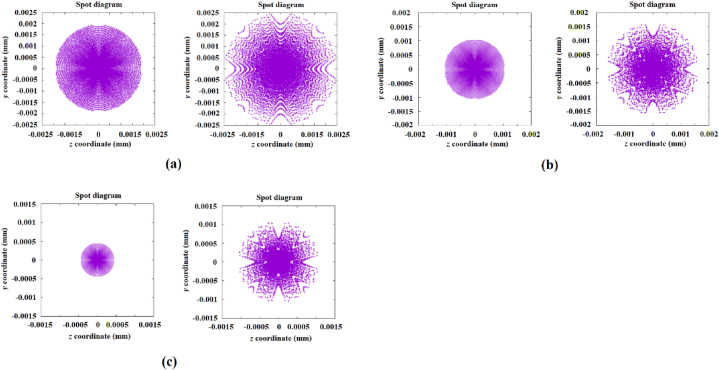
Comparison of the desired images (left) with the generated images reproduced by the designed mirrors (right) comprised of segmented rings with spherical profiles. The desired images correspond to the mirrors with diameters: a) 50, b) 40, and d) 30 mm.

The corresponding spot diagrams of these mirrors, together with the spot diagrams generated by the designed freeform mirrors with spherical segments and a constant diameter of 100 mm, are shown in Fig. 9, Fig. 10. In Fig. 9a, b, 9c and 9d are shown the desired images that correspond to the mirrors with diameters of 90, 80, 70, and 60 mm respectively. Whilst in Fig. 10a, b, and 10c are shown the desired images that correspond to the mirrors with diameters of 50, 40 and 30 mm, respectively. In each one of these figures are included the corresponding generated image.

The sum of the errors obtained with the aid of genetic algorithms of the generated images from the designed mirrors with diameters 90, 80, 70, 60, 50, 40 and 30 mm are 0.4914e-3, 0.2918e-3, 0.3369e-3, 0.4645e-3, 0.5753e-3, 0.6721e-3 and 0.7128e-3, respectively.

It can also be observed that the size and shape of the reproduced images from the mirrors with diameters of 60 to 30 mm are considerably different from their corresponding desired images. It is evident that the proposed design is practical when it comes to the concentration of energy, although the image's shape is lost. In order to generate images similar to the desired images, in the following section, a mirror design with a surface comprised of segmented rings with conic profiles is proposed.

### 3.5 Designs of mirrors with fixed diameter to generate images from mirrors with smaller diameters – segmented rings with a conic profile

Mirrors comprised of segmented rings with conic profiles are considered, as they are able to reproduce similar images in shape to the ones generated by the previously designed mirrors with diameters of 60, 50, 40, and 30 mm, respectively.

**Fig. 11:**
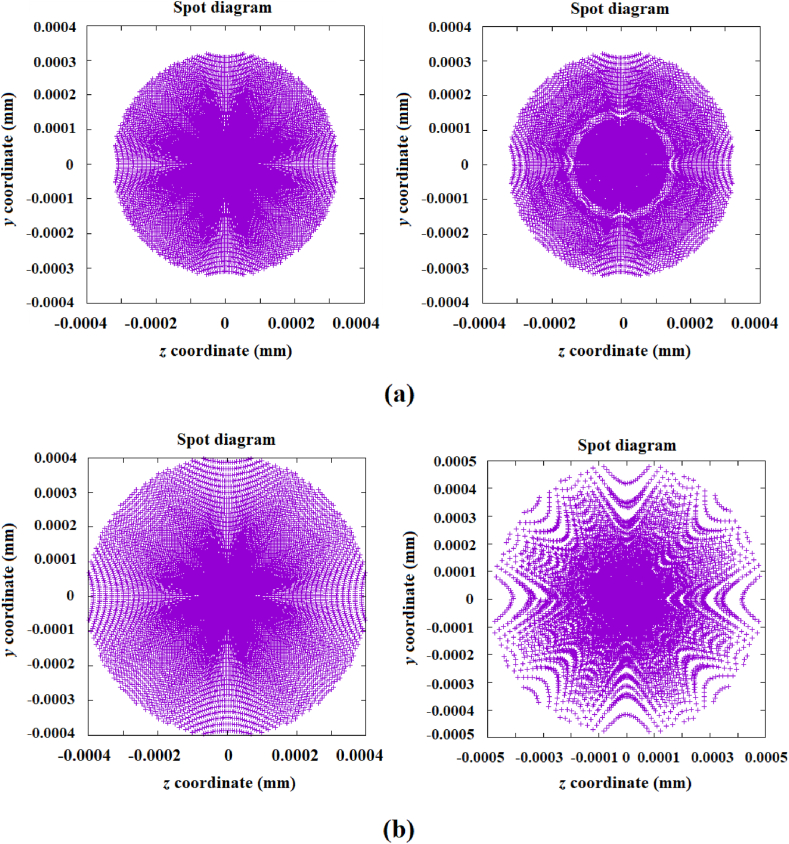
Images generated by the designed mirrors with a 100 mm diameter with a surface comprised of a set of segmented rings with conic profiles vs. images from spherical mirrors with a smaller diameter. a) Left: image to be reproduced, generated by a spherical mirror with a 60 mm diameter; right: image generated by the designed mirror; b) Left: image to be reproduced, generated by a spherical mirror with a 30 mm diameter; right: image generated by the designed mirror.

General ray trace equations for conic surfaces were employed for these designs [33]. In this case, the optimization method was implemented to the conic constants of each one of the segments, meaning that the number of variables to be optimized increased. Likewise, the optimal conic constants were obtained by applying a recurrence relation to reduce the variables to be optimized [30,31]. Fig. 11 shows the images generated by the reference mirrors with 60 and 30 mm in diameter, along with the images generated by the designed mirrors comprised of segmented rings with conic profiles.

As shown in Fig. 11 (right column), the images generated with the freeform mirrors comprised of segmented rings with conic profiles are improved up to a certain reduced diameter. The image generated by the 30 mm diameter mirror differs considerably from the reference image, although the energy concentration functionality is evident, with a difference of approximately 20% in size.

It is worth mentioning that there are previous works in the literature which are similar in their design method to the work presented in this paper. The methodology used in these works is called ASAS (annularly stitched aspheric surfaces) which consists of a rotationally symmetric optical surface containing various zones defined by functions of aspheric profiles [34,35]. The smoothness along the surface is demonstrated through different optimization techniques. To obtain a single ASAS, two practical methods are described [34], the first of which uses a non-sequential surface (NSS), while the other uses a user-defined surface (UDS). Optimization with NSS ensures that the different zones are connected smoothly. The other optimization strategy implements a user-defined stitched asphere by using the *C* programming language which later interacts with a commercial optical design software, and it facilitates the automatic connection with two different zones. Multiple examples of the implementation of this method to optical systems are presented, which include an objective lens for a BD system and an ultrawide angle catadioptric lens. Both works [34,35] describe the implementation of the ASAS method for specific applications. The evaluation of the performance of the design of both optical systems stated above includes assessments of the Modulation Transfer Function (MTF), beam synthesis propagation (BSP) analysis, and image acquisition.

In this work, a method that uses aspheric segmented concentric rings is presented. When implemented, a desired shape of the spot and a desired spot size can be obtained, and the use of segmented rings with an optimized conic surface, using a Genetic Algorithm (GA), facilitates the design of surfaces that generate a non-symmetric concentration of energy in the image plane. Therefore, the performance results were focused on the concentration of energy from mirrors rather than a frequency analysis. In order to make a comparison between our design method with the previous works [34,35] and highlight the differences, the following information is presented:

The optimization process applied to the design of the mirrors designed by a set of segmented rings with a conical profile (shown in Fig. 11) consisted of dividing the mirror into 20 rings in order to generate a more continuous and smoother surface. Of these 20 rings, only the first 18 were segmented and optimized (as the last two were closest to the center, their optimization for energy concentration had no practical effect).

Secondly, three rings were optimized consecutively at the same time, with a conic constant computed with the Genetic Algorithm, assigned to each ring (and consequently assigned to each of the 36 segments into which the ring is divided). For each ring, the GA calculates 10 variables, one for the calculation of the initial radius of curvature, and the remaining 9 variables for the calculation of nine increments of the radii of curvature. With these 10 optimized variables the 36 radii of curvature are calculated for the 36 segments into which the ring is divided.

Therefore, the number of variables optimized by the GA for three rings at once is 33 variables, meaning that a total of 108 segments and three conic constants are calculated, one for each ring. Once the first three rings have been optimized, the optimization of the next three rings continues, and this process continues until all 18 rings have been completed. The total number of optimized segments of the 18 rings is 648 plus 18 conic constants. As a feature of the developed optimization process, the sum of squared errors can be decreased slightly if a second iteration is carried out, but there are usually no further significant changes to the shape and size of the image.

According to the GA sequential optimization process of optimizing three rings at a time, it is possible to further increase the number of rings into which the surface is divided to ensure a continuous free-form surface, even with a smooth profile. These aspects were shown in the design of a mirror with concentric spherical rings [30], in which 100 rings were used to design the surface so that it was as continuous as possible, as validated by the design of the surface generated by the commercial program Zemax version EE [30].

Regarding the smoothness of the designed surfaces that generate the spot diagrams shown in Fig. 11(a) and (b), Table 1 shows, for example, the initial radii of curvature, the increments in the radii of curvature and the conic constants calculated by the GA of the designed mirror that generates the image 11 (b) for the first three rings. These results (with values very similar to those calculated for rings 4 to 18) show that the difference between the values for the initial radii between adjacent rings is a maximum difference of a few hundredths of a millimeter.

**Table 1:** Values of the initial radius, curvature radius increments $\Delta r_{j}\text{,}$ and conic constants computed with the Genetic Algorithm for the first three rings to design the spherical mirror with 30 mm in diameter of Fig. 11(b).

Parameter	Ring 1	Ring 2	Ring 3
Initial radius (mm)	−2040.0695	−2040.0680	−2040.0763
$\Delta r_{1}\;\left(mm\right)$	8.6545E-05	−8.2724E-05	−7.6605E-05
$\Delta r_{2}\;\left(mm\right)$	−7.5220E-05	3.0093E-05	4.9300E-05
$\Delta r_{3}\;\left(mm\right)$	4.8044E-05	−2.5690E-05	9.2419E-05
$\Delta r_{4}\;\left(mm\right)$	−8.9542E-05	−5.0967E-05	9.6765E-05
$\Delta r_{5}\;\left(mm\right)$	9.5480E-05	4.9210E-05	−7.0688E-05
$\Delta r_{6}\;\left(mm\right)$	2.1130E-05	8.4180E-05	4.1269E-05
$\Delta r_{7}\;\left(mm\right)$	−4.0387E-05	−8.9740E-05	9.6835E-05
$\Delta r_{8}\;\left(mm\right)$	−4.6042E-05	3.8055E-06	6.3351E-05
$\Delta r_{9}\;\left(mm\right)$	−5.2240E-05	−3.1115E-05	−8.3072E-05
Conic constant	−0.85399	−0.84461	−0.81264

While the radii of curvature calculated for the increments generate very small differences between adjacent segments of thousandths or ten thousandths of a millimeter, this difference in values is due to the recurrence equation used, as described by Eq. (3). Moreover, regarding the calculated values of the conic constants, it can be observed in Table 1 that the difference measured in the conic constants between the adjacent rings (and consequently between the adjacent radial segments) is between 4 or 5 hundredths. Therefore, it can be concluded that the designed surface presents a good degree of continuity and smoothness. In addition, as in any implemented optimization method, in the GA the continuity and smoothness of the designed surface can be guaranteed by restricting the search range of the optimal values in the GA.

For the manufacture of these designed mirrors, the process that can be applied is the one reported for the design, manufacture, and validation of polymeric optical components with aspheric profiles for applications in visual sciences [36]. A high-precision CNC machine was used to manufacture the aluminum molds used for the obtention of the optical components. With this in mind, this paper proposes the use of these types of machines focused on this specific application, as it has been shown that a high-precision CNC machine is able to obtain an aspheric profile with a satisfactory performance. Several parameters from the machining process have an effect on the smoothness of the finished profile, and they have to be taken into account for the obtention of these surfaces, e.g., cutting speed, spindle speed, feed rate, etc. The manufacture and validation of the examples presented in this work are beyond the reach of our current study. However, the authors recognize that it is an area of study that could potentially be explored in the near future.

**Fig. 12:**
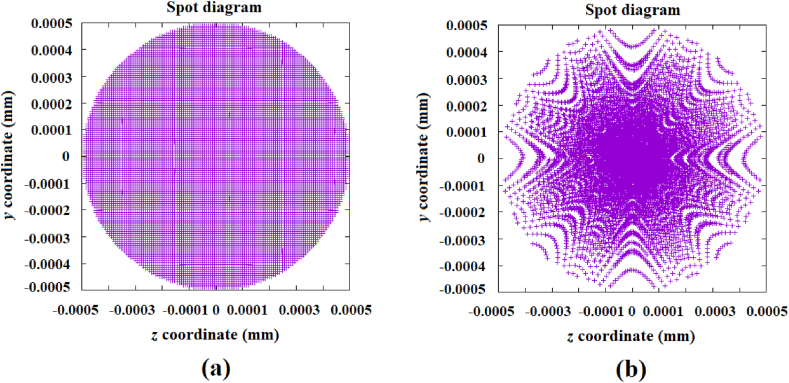
a) Image generated by a parabolic mirror with a diameter of 100 mm and a paraxial radius of curvature of 2040 mm, located at 1020.01 mm from the mirror's vertex. b) Image generated by the designed mirror with a 100 mm diameter, which reproduces the image from a mirror with a 30 mm diameter.

Finally, for the sake of comparison, Fig. 12a shows the generated image from a mirror with a parabolic profile of 100 mm in diameter and a paraxial radius of curvature of 2040 mm. The image is located at a distance of 1020.01 mm from the mirror's vertex (the Gaussian image is located at 1020.00 mm). In addition, Fig. 12b shows the image generated by the designed mirror comprised of segmented rings with conic profiles. The size of the reproduced image is comparable to the one generated by the parabolic mirror, as can be seen.

## 4 Discussion

As can be appreciated from the examples shown in the previous section, the proposed design methodology enables the designs of mirrors comprised of concentric segmented rings to reproduce images of a desirable size or similar to those produced by mirrors with smaller diameters as seen in section 3.1, where an image which has been reduced in size is presented. However, with this approach, a more significant concentration of energy is achieved.

On the other hand, this methodology also allows the design of segmented spherical mirrors to reproduce non-rotational symmetric images, as shown in the examples from sections 3.2 and 3.3. These features are required in specific applications that employ different types of apertures or a determined concentration of radiation.

Finally, the examples from sections 3.4 and 3.5 showed that the designed surfaces facilitate the reduction in size of the image when compared to the images produced by surfaces with smaller diameters. A reduction in optical aberrations is also implied, and the concentration of radiation is larger than the methodology carried out by surfaces with a smaller diameter. Therefore, this methodology can be adapted to the designs of several optical systems with potential applications in image formation, the concentration of energy, and illumination.

## 5 Conclusions

A brief review of the most used methods for the design of freeform surfaces found in the literature was presented along with their advantages. An alternative design methodology is also introduced that begins with the start design. When the radii of curvature and conic constants are optimized, aspheric or freeform surfaces are produced from a dish-shaped surface comprised of segmented concentric rings, producing the desired image sizes and shapes, as shown in the previous sections. However, the use of aspheric surfaces to produce the desired images with greater intensity is required in a wide variety of applications. Furthermore, it was shown that this design method allows the design of freeform surfaces to produce specific shapes and areas of energy concentration. Through the adequation of the merit function in the optimization process, the proposed method can be applied to the design of illumination, concentration, or imaging systems.

## Declarations

### Author contribution statement

Jorge González-García, Dr.: Conceived and designed the experiments; Performed the experiments; Analyzed and interpreted the data; Contributed reagents, materials, analysis tools or data; Wrote the paper.

Agustin Santiago-Alvarado, Dr.: Conceived and designed the experiments; Performed the experiments; Contributed reagents, materials, analysis tools or data.

Angel Sinue Cruz-Felix, Dr.: Conceived and designed the experiments; Performed the experiments; Wrote the paper.

### Funding statement

This research did not receive any specific grant from funding agencies in the public, commercial, or not-for-profit sectors.

### Data availability statement

Data included in article/supp. material/referenced in article.

### Declaration of interest’s statement

The authors declare no competing interests.
